# Retinal Neural and Vascular Structure in Isolated Growth Hormone Deficiency Children and Evaluation of Growth Hormone Treatment Effect

**DOI:** 10.4274/jcrpe.4758

**Published:** 2018-05-18

**Authors:** Özge Yüce, Nuriye Gökçen Yalçın, Aysun Bideci, Esra Döğer, Hamdi Cihan Emeksiz, Murat Hasanreisoğlu, Zeynep Aktaş, Orhun Çamurdan, Peyami Cinaz

**Affiliations:** 1 Gazi University Faculty of Medicine, Department of Pediatric Endocrinology, Ankara, Turkey; 2 Gazi University Faculty of Medicine, Department of Ophthalmology, Ankara, Turkey

**Keywords:** Growth hormone deficiency, retinal neural development, retinal vascularization, growth hormone treatment

## Abstract

**Objective:**

To evaluate neural and vascular retinal morphology of children with isolated growth hormone deficiency (GHD) and to determine any retinal changes due to GH treatment.

**Methods:**

Twenty-eight children with isolated GHD and 53 age-, gender- and body mass index-matched healthy volunteers were enrolled in this prospective study. The retinal nerve fibre layer (RNFL) and macular thickness (MT) were measured, as well as intraocular pressure (IOP). The number of retinal vascular branching points were calculated. Effect of GH treatment on the retina and IOP was evaluated after one year of treatment. Measurements were also made in the control group at baseline and following the initial examination. Pre- and post-treatment changes were compared. The findings were also compared with those of the controls. The correlation between ocular dimensions and insulin-like growth factor-I (IGF-1) levels were also analysed.

**Results:**

The number of branching points was significantly lower in GHD patients as compared with control subjects (15.11±2.67 and 19.70±3.37, respectively, p=0.05 for all comparisons). No statistically significant differences were found in mean RNFL, MT and IOP values between GHD patients and control subjects. GH treatment did not create any significant changes in the retinal vascularization or other retinal neural parameters and IOP either within the patient group or when compared with the control group. No correlations were observed between ocular dimensions and IGF-1 levels.

**Conclusion:**

Our findings suggest that isolated GHD may lead to decreased retinal vascularization. However, retinal neural growth and differentiation were not affected by GHD. These findings may be related to the fetal development process of pituitary somatotropic cells and the retina. Additionally, GH treatment did not cause any changes in retinal neural and vascular tissues.

## What is already known on this topic?

Reduced retinal vasculature has been shown in patients with growth hormone (GH) deficiency and insensitivity. However, retinal neural morphology in these patients evaluated in different studies, reported different results. Unlike retinal vascularization, data regarding retinal neural structure are discrepant between different studies. Some studies reported increased, while others reported decreased retinal nerve fibre layer thickness or macular thickness. Additionally, different parameters have been used in the limited number of studies evaluating the effect of GH treatment on ocular tissues.

## What is this study adds?

Our findings suggest that GH deficiency may lead to decreased retinal vascularization. However, retinal neural growth and differentiation were not affected by GH deficiency. We also evaluated the effect of GH treatment on the retina and observed that GH treatment did not cause any retinal changes.

## Introduction

Growth hormone (GH) is released from pituitary somatotrophs into the circulation and is essential for postnatal growth and development. Experimental models have demonstrated the presence and effect of pituitary GH in many extrapituitary sites, including the nervous, reproductive, immune and vascular systems (1,2). The ocular neural and vascular system is one of these sites.

The possible role and effect of pituitary GH and subsequently generated insulin-like growth factor-1 (IGF-1) on retinal development is controversial. However, a few recent human studies have emphasized its functional role. Although GH and various growth factors (IGFs, vascular endothelial growth factor, fibroblast growth factor and transforming growth factor-beta) are often thought to be produced locally and act in autocrine/paracrine ways to promote the maintenance, survival and differentiation of retinal tissues, this is only partially true for the vascularization and neurogenesis of the retina before the functional differentiation of pituitary somatotrophs (3).

Abnormal ocular findings such as optic nerve hypoplasia, disc dysfunction, increased corneal thickness, reduced retinal vascularization and short axial length in GHD patients demonstrate the effect of reduced GH production on the developing ocular tissues (4,5,6,7,8). Presence of pituitary GH in the human retina and vitreous fluid also provides further evidence for a possible role of GH in ocular development (9,10). Consequently, ocular tissues seem to represent a target site for pituitary GH action, as suggested by several human and animal studies. Based on these studies, we aimed to evaluate retinal neural and vascular structure in isolated GHD patients. Another objective of this study was to assess any retinal changes developing as a result of GH treatment.

## Methods

This prospective study consisted of 28 patients with severe short stature (height standard deviation score less than -3) at diagnosis and whose growth velocity was lower than 4 cm/year or below expectations for the pubertal stage. Patients were excluded if they had a history of being preterm or small for gestational age at birth; a history of cardiovascular, thyroid, hepatic or renal disease or obesity; current hypertension, chromosomal abnormalities or, in addition to their already known ocular disease, had severe refractive errors or a family history of ocular hypertension/glaucoma. 

Pubertal staging was assessed by Tanner stage according to breast development in girls and genital development in boys (11). Routine biochemical tests, complete blood counts, thyroid function tests and serum tissue transglutaminase antibodies were obtained in all patients. Bone age was evaluated by using the Greulich and Pyle atlas. Pituitary magnetic resonance imaging (MRI) was performed in all to exclude presence of a structural anomaly. All patients underwent a GH stimulation test performed with L-Dopa and Clonidine. IGF-1 and IGF-binding protein-3 (IGFBP-3) levels were also measured. A peak GH responses below 10 µg/L after two stimulation tests was accepted as GH deficiency. The diagnosis of GHD was confirmed according to the clinical, auxological and biochemical criteria of the Growth Hormone Research Society (12). In diagnosed patients, recombinant human GH treatment was started at an initial daily dose of 0.025 mg/kg. During the study, IGF-1 and IGFBP-3 levels were measured at intervals of 3-6 months and the GH dose was adjusted to maintain serum IGF-1 levels above + 2 standard deviation (SD), but not exceeding + 3 SD levels.

Fifty-three healthy children who were carefully matched for age, gender, body mass index (BMI) and pubertal staging were recruited as the control group from the siblings of patients and children who had presented to the health care unit for a routine examination. Healthy volunteers were recalled at the end of the first year following the initial selection. The study protocol was approved by the ethics committee of Gazi University, Faculty of Medicine (approval number: 357-14/07/2014). Parents of the patients and controls were informed about the study and informed consent was obtained.

### Assessment of Retinal Vascularization and Retinal Thickness

All patients and controls underwent a complete ophthalmologic examination, including an auto-refractometer (RM8900; Topcon), best-corrected visual acuity measurements with a 6 meter Snellen eye chart, slit-lamp biomicroscopy, fundus examination and intraocular pressure (IOP) measurement. The subjects also underwent an examination with the Heidelberg Spectralis-OCT (Spectralis; Heidelberg Engineering, Heidelberg, Germany). Central subfield macular thickness (MT) and peripapillary retinal nerve fibre layer (RNFL) thickness were assessed to evaluate retinal neurogenesis. The RNFL measurements were determined globally and for six regions including temporal, supertemporal, supernasal, nasal, inferotemporal and inferonasal regions. The number of retinal vascular branching points was obtained from infrared images. Optic nerve head (ONH) centered near-infrared reflectance pictures were obtained with a 30° field of view. A frame to calculate retinal vascular branching points was set for each eye to minimize individual disparity. Temporal border of the fovea was defined as the temporal border of the frame. The other borders were calculated as 4000 µm away from the center of the ONH.

RNFL thickness, MT and retinal vascularity were evaluated again after 12 months of treatment and any retinal IOP changes, were recorded. Additionally, the correlation of changes with IGF-1 levels were calculated. The controls were also called back one year following their initial examination to record any retinal changes associated with age and puberty.

### Statistical Analysis

All statistical calculations were performed using SPSS version 20 (SPSS, IBM Inc., Chicago). The subjects’ right eyes were selected for statistical analysis. Descriptive statistics were computed as means ± SD. Parameters with normal distribution were analysed with the t-test and parameters with non-normal distribution with the Mann-Whitney U test. Differences between values before and after GH treatment were evaluated using paired samples t-tests. The linear relationships between variables were evaluated using Pearson’s correlation tests. A p value <0.05 was considered statistically significant.

## Results

A total of 28 (female/male ratio: 14:14) isolated GHD patients and 53 (female/male ratio: 33:20) age- and gender-matched healthy children aged 12.46±2.41 and 11.32±3.1 years, respectively, were included in the study. No statistical difference was observed among the groups in terms of age, gender or BMI (p>0.05). Puberty was compatible with Tanner stage 1 in nine patients and in 19 control subjects (p>0.05). Tanner stage 2 and 3 occurred in the remaining subjects (8 patients and 15 control subjects in stage 2; 11 patients and 19 control subjects in stage 3). Routine biochemical tests, complete blood counts, thyroid function tests and serum tissue transglutaminase antibodies were within normal limits. Pituitary MRI was normal in all patients.

**Figure 1:**
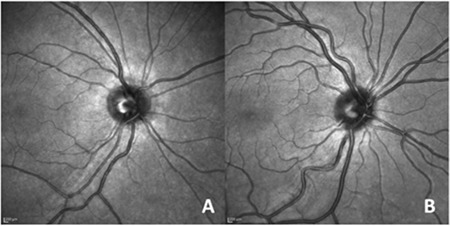
Infrared images; A) reduced retinal vascularization in a 10-year-old growth hormone deficiency patient, B) normal retinal vascularization in a 10-year-old healthy subject

The best corrected visual acuities of all subjects were 6/6. None of the differences in spherical equivalent, MT and four quadrant RNFL thickness, IOP among patients and control subjects were significant (p>0.05). The mean number of vascular branching points was 15.11±2.67 in the study group and 19.70±3.37 in the control group (p<0.01, Figure 1). All ocular parameters of both groups are compared in Table 1. 

At the end of the first year, eye parameters of all 28 patients were checked. No significant changes were observed in the MT, RNFL thickness, IOP and the number of vascular branching points after treatment (p>0.05; Table 2). In one GHD patient, optic disc drusen were detected and the patient was followed.

Additionally, retinal vascularization, IOP, MT and RNFL thickness did not show any significant correlation with an increase in IGF-1 levels (r= 0.003; r= 0.12; r=-0.06; r=0.16, respectively, p>0.05 for all). 

The first-year evaluation of the control group could be performed in only 17 subjects. No significant differences in ocular parameters related to pubertal development and age were observed in these subjects. The stastistical results of the initial comparison were not different from the statistical results of the second comparison in which we compared the retinal measurements of the patients that were under treatment with GH and the controls. 

## Discussion

In this prospective study, we have essentially examined the retinal neural and vascular structures in patients with isolated GHD to explore whether GH treatment will cause any retinal change. We observed that the MT and RNFL thickness of patients was not different from healthy controls, while retinal vascularization decreased. On the other hand, GH treatment did not cause any retinal neuro-vascular changes.

Hellström et al (7) first reported reduced retinal vasculature in isolated GHD patients. These authors also drew attention to the importance of GH and IGF-1 for normal retinal vascularization. More recently, Pereira-Gurgel et al (13) reported moderate reduction of retinal vascular branching points in isolated GHD patients. Another recent study also evaluated the effects of the GH/IGF-1 axis on retinal vascular branching and other characteristics in patients with GH insensitivity and reported reduced retinal vasculature and tortuosity of the retinal vessels (14). Similarly, our patients also showed reduced retinal vasculature. Based on these data, we suggest that the GH/IGF-1 axis has an effect on retinal vasculature. However, we cannot show that pituitary GH has an effect on retinal neural development. In fact, GH and GH mRNA proteins have also been identified in retinal ganglion cells and can be traced within their axons in the retina within the optic fibre layer and outside the retina within the optic nerve, optic chiasm and optic tract (10,15,16). In view of this knowledge, we expected that the GHD would have led to greater thinning of the MT and RNFL thickness, but our findings did not support this expectation. Decreased peripapillary nerve fiber thickness and decreased optic disc size were previously described in some children with congenital GHD (17,18). The differences between these reports and our findings are probably due to the variety of causes of GHD as well as to differences in methodology.

The reason why retinal neural structure shows normal development while retinal vascularization is decreased in GH deficiency can be partially explained by the embryonic development process of these structures and somatotrop cells. GH producing cells can be identified at nine weeks of gestation (19), while embryonic neural development occurs in an earlier period. Therefore, it can be assumed that the development of the nervous system in its early stages is independent of GH and shows normal development, despite the lack of pituitary GH, or that its development is affected by GH produced in an extra pituitary site. Our findings also suggest that locally produced GH and factors are more effective in neural retinal development. This possibility is supported by the presence of GH immunoreactivity in the brain prior to the ontogeny of the pituitary gland and somatotroph differentiation, as has been demonstrated in the human and chicken brain (20,21). Vascularization of the retina normally starts at approximately 12 weeks of gestation, while pituitary somatotroph GH production has already begun and continues during fetal development, with little or no vascularization after birth (22,23). Normally, it is accepted that IGF-2 has a greater effect when fetal somatic and ocular development is considered. However, the effect of GH and IGF-1 on retinal vessels cannot be ignored. This hypothesis is supported by several studies showing a decrease in retinal vascularization in patients with GHD and GH insensitivity (24).

Another finding of this study was that GH treatment did not create any significant retinal changes, nor changes in IOP. The effect of a lack of effect of GH treatment on the retina can be explained in various ways. One proposal suggests that GH and IGF-1 cannot pass from the inner retinal barrier (25). Other proposals suggest that normalized GH and IGF-1 levels might not be sufficient to lead to significant retinal changes or that the follow-up time may be inadequate to evaluate retinal changes (26). 

Previous data suggesting induction of neovascularization by IGF-1 following investigation of clinical conditions where an excess of IGF-1 is present in the serum (27,28,29,30). Treatment with GH, on the other hand, aims to normalize IGF-1 levels as much as possible and is not expected to induce a sustained excess of IGF-1, as in acromegaly or in patients with diabetic retinopathy. Another explanation may also be related to the impairment of the retinal blood barrier permeability and/or impairment of integrity in diabetic retinopathy.

### Study Limitations

Our study has some limitations. As was the case with other similar studies, the lack of a genetic diagnosis was the most significant limitation. Another limitation was the use of a semi-quantitative system to assess the retinal vascularization. Long-term treatment outcomes should be evaluated to determine correct and more extensive information about the effect of HGH treatment on the retina and this was also a potential limitation of our study. 

## Conclusion

The best of our knowledge, the current study is the first comprehensive prospective study to evaluate retinal structure (neural and vascular) in isolated GHD children. A selective reduction of retinal vascularization and normal retinal neural architecture may suggest that the GH/IGF-1 axis regulates retinal vascular development, but not the neural retina. Besides, our findings suggested that GH treatment is not associated with retinal changes. However, monitoring time, treatment dose and the etiology of GHD should be taken into consideration when stating that HGH treatment has no effect on ocular parameters. We recommend, therefore, that ophthalmologic evaluations should be performed in all GHD patients before institution of GH treatment and that these be repeated annually. Also, further studies with larger groups are required to clarify the functional role of GH on ocular growth and differentiation by using advanced measurement techniques.

## Figures and Tables

**Table 1:**
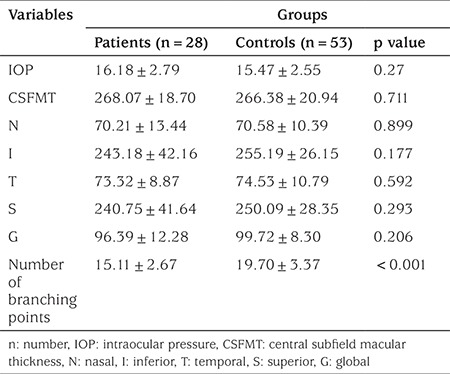
Ocular parameters of patient and control groups at baseline

**Table 2:**
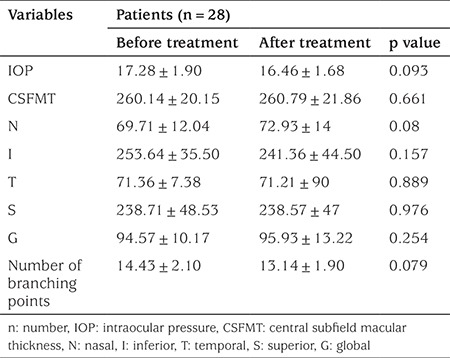
Ocular parameters of patients before and after 1-year of growth hormone treatment
